# Noonan syndrome

**DOI:** 10.1186/1750-1172-2-4

**Published:** 2007-01-14

**Authors:** Ineke van der Burgt

**Affiliations:** 1 Department of Human Genetics, University Medical Centre st Radboud, PO Box 9101, 6500 HB Nijmegen, The Netherlands

## Abstract

Noonan Syndrome (NS) is characterised by short stature, typical facial dysmorphology and congenital heart defects. The incidence of NS is estimated to be between 1:1000 and 1:2500 live births. The main facial features of NS are hypertelorism with down-slanting palpebral fissures, ptosis and low-set posteriorly rotated ears with a thickened helix. The cardiovascular defects most commonly associated with this condition are pulmonary stenosis and hypertrophic cardiomyopathy. Other associated features are webbed neck, chest deformity, mild intellectual deficit, cryptorchidism, poor feeding in infancy, bleeding tendency and lymphatic dysplasias. The syndrome is transmitted as an autosomal dominant trait. In approximately 50% of cases, the disease is caused by missense mutations in the *PTPN11 *gene on chromosome 12, resulting in a gain of function of the non-receptor protein tyrosine phosphatase SHP-2 protein. Recently, mutations in the *KRAS *gene have been identified in a small proportion of patients with NS. A DNA test for mutation analysis can be carried out on blood, chorionic villi and amniotic fluid samples. NS should be considered in all foetuses with polyhydramnion, pleural effusions, oedema and increased nuchal fluid with a normal karyotype. With special care and counselling, the majority of children with NS will grow up and function normally in the adult world. Management should address feeding problems in early childhood, evaluation of cardiac function and assessment of growth and motor development. Physiotherapy and/or speech therapy should be offered if indicated. A complete eye examination and hearing evaluation should be performed during the first few years of schooling. Preoperative coagulation studies are indicated. Signs and symptoms lessen with age and most adults with NS do not require special medical care.

## Disease name and synonyms

Noonan syndrome (NS)

(Mistakenly: male Turner syndrome, Turner phenotype with normal karyotype, female Turner-like syndrome)

## Definition and diagnostic criteria

Noonan Syndrome (NS) is an autosomal dominant disorder characterised by short stature, typical face dysmorphology and congenital heart defects. NS is a clinical diagnosis. Establishing the diagnosis can be very difficult, especially in adulthood. There is a great variability in expression and the phenotype becomes less pronounced with increasing age [1]. Several scoring systems have been devised to help the diagnostic process. The most recent scoring system was developed in 1994 [2] (Table 1).

**Table 1:** Scoring system for Noonan syndrome (NS) #

**Feature**	**A = Major**	**B = Minor**
1 Facial	Typical face dysmorphology	Suggestive face dysmorphology
2 Cardiac	Pulmonary valve stenosis, HOCM and/or ECG typical of NS	Other defect
3 Height	<P3*	<P10*
4 Chest wall	Pectus carinatum/excavatum	Broad thorax
5 Family history	First degree relative with definite NS	First degree relative with suggestive NS
6 Other	Mental retardation, cryptorchidism and lymphatic dysplasia	One of mental retardation, cryptorchidism, lymphatic dysplasia

HOCM: hypertrophic obstructive cardiomyopathy;

*P3 and P10 refer to percentile lines for height according to age, with the normal range of variation defined as P3-P97 inclusive

Definitive NS: 1 "A" plus one other major sign or two minor signs; 1 "B" plus two major signs or three minor signs

# adapted from [2]

## Epidemiology

The incidence of NS is reported to be between 1 in 1000 and 1 in 2500 live births [3,4].

## Clinical description

Common features of NS include:

### Characteristic facial features that change with age

In the postnatal period the forehead is broad and high, there is hypertelorism, epicanthic folds and downward slanting palpebral fissures, low-set posteriorly rotated ears with a thick helix, high arched palate, micrognathia, and a short neck with excess nuchal skin and a low posterior hairline. The contour of the face becomes more triangular with age and in childhood the face often appears coarse or myopathic, with prominent eyes and (unilateral or bilateral) ptosis, and thick lips with prominent nasolabial folds. In the adolescent and young adult, the eyes are less prominent and the neck appears less short. Sometimes there is marked webbing or prominent trapezius. Typically, older adults have prominent nasolabial folds, a high anterior hair line, thick hooded eyelids and wrinkled skin [1,4]. The facial features can be subtle, especially at old age.

The most common congenital **heart defect **is pulmonary valve stenosis with dysplastic leaflets (50%–62%) [5,6]. Hypertrophic obstructive cardiomyopathy (HOCM) with asymmetrical septum hypertrophy is present in 20% of patients. Atrial septal defects occur in 6%–10% of cases, ventricular septal defects occur in 5% of cases and persistent ductus arteriosus occurs in 3% of cases [3,4]. Other congenital heart defects more often seen in NS are atrioventricular canal defect (AVCD) associated with subaortic obstruction and structural anomalies of the mitral valve [7].

Electrocardiograms (ECG) from NS patients display wide QRS complexes with a predominantly negative pattern in the left precordial leads (62%). They also display left axis deviation and giant Q waves [5,8].

**Weight and length **are usually normal at birth. Birth weight can be high due to subcutaneous oedema. In such cases, marked weight loss occurs in the first week of life. Neonatal feeding difficulties and failure to thrive are present in 63% of patients [4]. In general, these feeding problems resolve spontaneously later in infancy. Mean prepubertal growth parallels the 3rd centile for height and weight. Onset of puberty is delayed by approximately two years and the pubertal growth spurt is often reduced or absent. The average bone age is also delayed by two years. Mean adult height is 162.5 cm in males and 152.7 cm in females. Both values are below the 3rd centile. Noonan-specific growth curves were designed in 1988 [9]. Growth hormone (GH) levels are in the normal range. Somatomedin levels are elevated in some cases. GH treatment in pharmacological doses can be used to accelerate growth during the first years of life. Initial reports on the long-term effects of this treatment show a beneficial effect [10,11]. NS patients with a mutation in the *PTPN11 *gene respond less efficiently to GH than NS patients without a mutation in *PTPN11 *[12].

Characteristic **chest deformities **consist of pectus carinatum superiorly and pectus excavatum inferiorly. These sternal abnormalities are present in 70%–95% of cases. The thorax is broad and the internipple distance is large. About 15% of patients develop thoracic scoliosis. The shoulders are often rounded with scapula alata. Other common orthopaedic features include cubitus valgus (50%), radioulnar synostosis (2%), clinobrachydactyly (30%), joint hyperextensibility (50%) and talipes equinovarus (12%) [3,4]. Giant cell lesions of the jaw, similar to those seen in cherubism, have been reported in several patients [13].

**Undescended testicles **at birth are common in male patients (77%). Increased luteinizing hormon (LH) and follicle stimulating hormone (FSH) levels are present in prepubertal boys [14]. High FSH levels and poor quality semen have been found in adults, suggesting a failure of spermatogenesis in patients with testicular maldescent [15]. In both sexes, pubertal development is delayed. The mean age of menarche in female patients is 14.6 years [4]. However, fertility is not impaired in females with NS.

**Urinary tract malformations **are present in 10% of cases, mostly pyelo-ureteric stenosis and/or hydronephrosis [4,16].

**Increased bruising or bleeding **is frequent, especially in childhood. Up to 55% of cases have a mild-to-moderate bleeding tendency. Severe haemorrhage occurs in 3% of cases. Coagulation studies reveal prolonged bleeding times, factor VIII, XI and XII deficiencies, thrombocytopaenia and platelet function defects. These manifestations appear individually or in combination [4,17]. For NS patients, there is no correlation between the results of coagulation tests and a history of easy bruising. Thus, these tests may not be predictive of bleeding risk in NS patients. Since many patients undergo one or more operations, special care is required to prevent intraoperative or postoperative haemorrhage. Suitable blood products should be made readily accessible in case such complications arise [18].

**Acute leukaemia and myeloproliferative disorders **(MPD) have been described in some patients. The 218C>T mutation in the *PTPN11 *gene is associated with a predisposition to an MPD, which most often resolve spontaneously [19]. In rare cases, individuals with NS can develop fatal MPD, typically juvenile myelomonocytic leukaemia (JMML). However, the prognosis for NS patients with JMML is better than that for non-NS patients with JMML [19,20].

**Lymphatic vessel dysplasia, hypoplasia, or aplasia **are common findings in NS (20%). They lead to generalised lymphoedema, peripheral lymphoedema, pulmonary lymphangiectasia or intestinal lymphangiectasia. The most common manifestation is dorsal limb lymphoedema, which usually disappears during childhood. Varying degrees of oedema or hydrops are present during intrauterine life. Ultrasound examination may reveal a cystic hygroma in early pregnancy. This swelling subsequently regresses and results in excess nuchal skin and pterygium colli after birth. Other features, which may be due to disruption of normal tissue migration or organ placement by foetal oedema, are cryptorchidism, wide-spaced nipples, low-set posteriorly rotated ears, hypertelorism and downward slanting palpebral fissures [21,22]. Spontaneous chylothorax may occur in childhood and chylous effusion is a known complication of cardiac surgery and surgery for thoracic deformity [23,24].

**Abnormalities of pigmentation **in NS include pigmented naevi (25%), *cafe-au-lait *spots (10%) and lentigines (3%). Ulerythema ophryogenes (*keratosis pilaris atrophicans faciei*) is present in 14% of cases and may lead to a lack of eyebrows. NS is also often accompanied by keratosis pilaris on the upper arms [4,25]. Approximately one-third of the patients have thick curly hair, and 10% have thin sparse hair. Foetal pads on fingers and toes are common (67%) [4].

Frequent **ophthalmic abnormalities **are strabismus (48%–63%), refractive errors (61%) and amblyopia (33%). Anterior segment changes (63%) and fundal abnormalities (20%) may be present, nystagmus is seen in 10% of NS patients [26,27].

**Hearing loss **due to otitis media is a frequent complication (15%–40%). Sensorineural hearing loss is less common, but involves the low frequency range in 10% of patients and the high frequency range in 25% of patients [28]. Structural anomalies of the inner ear have occasionally been reported [29,30] and vestibular abnormalities have been described in a single case [31].

**Hepatosplenomegaly **unrelated to cardiac failure is often present in infancy (26%–51%). It is less frequent with higher age. There are no reports of hepatic and/or splenal dysfunctions being associated with the organomegaly [4,19].

In general, children with NS demonstrate **mild motor delay**, which may be partly attributed to the muscular hypotony that is often present in early childhood. Mean age for sitting is 10 months, that for walking alone is 21 months, and that for talking is 31 months [4]. Articulation abnormalities are frequent (72%). Mental retardation is present in 15%–35% of cases and is usually mild. It is characterised by specific visual-constructional problems and verbal performance discrepancy. Mean full scale intelligence quotient (IQ) is 85, but there is a wide range in the level of intelligence [32,33]. Prominent behavioural problems are clumsiness, eating problems, fidgety or stubborn spells, echolalia and irritability. Social problems and attention deficit have also been noted. In spite of this wide range of reported behavioural problems, most authors state that children with NS can be raised with parental support alone, without any specialised intervention [34].

## Aetiology

NS may occur on a sporadic basis or in a pattern consistent with autosomal dominant inheritance with a predominance of maternal transmission.

In approximately 50% of the patients with definite NS, a missense mutation is found in the *PTPN11 *gene on chromosome 12. *PTPN11 *encodes the non-receptor protein tyrosine phosphatase SHP-2. This enzyme is involved in a wide variety of intracellular signal cascades downstream of receptors for growth factors, cytokines and hormones, and is required in several developmental processes [35]. The mutations associated with NS result in a gain of function of SHP-2 [36].

Heterogeneous gain-of-function mutations in *PTPN11 *are found in almost half of the patients with clinically definite NS. These mutations are found in 59% of the familial cases and in 37% of the sporadic cases. Most of the mutations are recurrent and cluster in exons 3, 8 and 13. Among individuals with NS, those with a mutation in *PTPN11 *more often have pulmonary valve stenosis, while those without a mutation in *PTPN11 *more often have a cardiomyopathy The most frequent NS-causing *PTPN11 *mutation is an A-to-G transition at nucleotide 922. This transition accounts for 25% of all NS-causing *PTPN11 *mutations and leads to a Asn308Asp substitution. This mutation is often found in familial cases and is not associated with any developmental abnormality. A genotype-phenotype correlation has also been identified between a C-to-T transition on nucleotide 218 (Thr73Ile) and a predisposition to a myeloproliferative disorder. This disorder most often resolves spontaneously [19,20,36]. Recently activating mutations in the *KRAS *gene, which is associated with the Ras pathway, were found as the causative dominant mutations in a few cases with NS. These findings establish hyperactive Ras as a cause of developmental abnormalities seen in NS [37].

## Diagnostic methods

Noonan syndrome is a heterogeneous but clinically recognisable, multiple congenital anomaly syndrome. Scoring systems can help the diagnostic process [2]. NS mostly occurs on a sporadic basis or in a pattern consistent with autosomal dominant inheritance, with a predominance of maternal transmission [3,4,19,20]. The *de novo PTPN11 *mutation in sporadic NS cases is predominantly of paternal origin [38]. However, there is evidence for a rare autosomal recessive form of NS [39].

## Differential diagnosis

There are a number of conditions with phenotypes strikingly similar to NS. The first to mention is Turner syndrome (45, X0), a well known chromosomal abnormality in girls. Then there are a group of distinct syndromes with partially overlapping phenotypes in which causative mutations are found in genes of the RAS-MAPK pathway. These include Cardio-Facio-Cutaneous (CFC) syndrome, Costello syndrome, Neurofibromatosis type 1 (NF1) and LEOPARD syndrome (multiple lentigines, ECG conduction abnormalities, ocular hypertelorism, pulmonic stenosis, abnormal genitalia, retardation of growth and deafness). Individuals with LEOPARD syndrome may have distinct mutations in *PTPN11 *wich lead to a diminished catalytic activity of these SHP-2 mutants. Costello syndrome is caused by mutations in *HRAS*, NF1 by mutations in *Neurofibromin *and CFC syndrome by mutations in *BRAF*, *KRAS *and *MEK1/2 *[40,41].

Syndromes that are characterised by facial dysmorphology, short stature and cardiac defects may sometimes be difficult to differentiate from NS, notably Williams syndrome and Aarskog syndrome [42].

## Genetic counselling

Before the child is born, consultations with parents may address the following, as appropriate:

• Explain the mechanisms for occurrence or recurrence of NS in the foetus and the recurrence risk in the family.

• Review the natural history and manifestations of NS, including variability.

• Discuss further studies that should be done, particularly those in the newborn period that will confirm the diagnosis. If miscarriage, stillbirth or termination occurs, confirmation of the clinical diagnosis by autopsy is also important.

• Review the currently available treatments and interventions.

• Explore the options available to the family for the management and rearing of the child.

## Antenatal diagnosis

NS should be considered in all foetuses with polyhydramnion, pleural effusions, oedema and increased nuchal fluid with a normal karyotype [21,43]. If there is clinical evidence of NS in the foetus or a first-degree relative has NS, obstetric ultrasound is indicated at 12–14 and 20 weeks' gestation and again in the third trimester. Foetal echocardiography is indicated at 18–20 weeks' gestation. If NS is suspected in the unborn child, physical examination of the parents for features of the syndrome is indicated. As normalisation of the facial phenotype with age is common in NS, a review of childhood photographs of both parents is often helpful. A DNA test for mutation analysis can be carried out on blood, chorionic villi and amniotic fluid samples. Herewith also preimplantation genetic diagnosis becomes a possibility.

## Management including treatment

The majority of children with NS will grow up and function normally in the adult world. However, they need special care and counselling. Below is a set of guidelines designed to assist physicians caring for NS patients and their families. Familiarity with the characteristic features of NS is clearly important for clinical geneticists, cardiologists, surgeons, anaesthetists, gynaecologists, paediatricians and dermatologists. Issues that need to be addressed at a given age are discussed.

### Issues that need to be addressed

#### From birth to 1 month – newborns

• Confirm diagnosis.

• Extensive cardiological examination including echocardiography.

• Appropriate laboratory studies, including chromosome analysis and DNA analysis (*PTPN11, KRAS*) if possible.

• Document measurements.

• Hepatosplenomegaly?

• Undescended testes in male patients? Initiate treatment if present.

• Weight loss in the first week.

• Hypotonia, poor feeding and failure to thrive.

• Offer extensive genetic counselling to the parents.

#### From 1 month to 1 year – infancy

• Growth and development.

• Serous otitis media.

• Cardiologic evaluation.

• Feeding and feeding difficulties.

• Support available to the family.

• Motor development (expect mild motor delay in most, but significant psychomotor delay in only a minority of patients).

#### From 1 to 5 years – early childhood

• Growth and development.

• Cardiologic evaluation.

• Speech.

• Easy bruising/coagulation.

• Cutaneous findings.

• Partial growth hormone deficiency?

• Possibility of growth hormone therapy in very small NS children with partial growth hormone deficiency.

• Behaviour and possible behavioural problems.

#### From 5 to 13 years – late childhood

• Social adaptation.

• Skeletal age.

• Growth hormone therapy if indicated.

• Cardiologic evaluation.

• School readiness/intellectual capabilities.

• Vision and hearing.

• Delay in puberty (on average about 2 years).

• Contact with other patients (especially valuable at this age)

• School performance.

#### From 13 to 21 years or older – adolescence to early adulthood

• Auxological parameters.

• Cardiologic evaluation.

• Coagulation (bleeding abnormalities present in childhood often resolve with age).

• Growth hormone therapy (if indicated) until adult height is reached.

• School performance and choice of profession.

• Genetic counselling at adolescent or young adult age.

## Prognosis

New medical problems are not expected to appear in adulthood. However, males who were born with undescended testes may have fertility problems. There is no evidence for gynaecological or childbearing complications in females with NS. Some patients have health problems as a consequence of their congenital heart defect, lymphatic vessel dysplasia, urinary tract malformation, haematological disorder, or other anomaly associated with NS. Nonetheless, most adults with NS do not require special medical care.

## Unresolved questions

It is clear that there is wide variability in the phenotypic expression of NS and that there are many unresolved questions. The phenotype varies from adults with mild facial features and a minimal pulmonary valve stenosis to severe dysmorphisms plus life-threatening heart disease in neonates. Furthermore, there is a wide variability in the intellectual and adaptive behaviour. It is not clear from where this wide variability in symptoms stems. Genotype-phenotype correlation studies among individuals with NS caused by a mutation in *PTPN11 *give some insights. The finding of mutations in one of the key genes in the RAS pathway (*KRAS*) in some patients with NS, however, rises a lot of new questions. There are overlapping features with Costello and CFC syndrome in NS, but there are also very different features present in these clinically distinct syndromes. The finding of germline gain-of-function mutations in genes from the RAS-MAPK pathway in NS, Costello and CFC syndrome unexpectedly assigns roles in human development to genes, which are much better known for their effects on dysregulation of cell signalling in cancer cells.

The aetiology of NS in individuals without mutations in *PTPN11 *or *KRAS *(almost 50% of cases) is still unknown. Research aimed at identifying the other gene(s) responsible for NS is ongoing.

Other unexplained findings of interest are: the near absence of large families with NS, the fact that all cohorts in studies of NS included more male than female patients, and why results of coagulation tests are not predictive of the bleeding risk in people with NS.
